# Determinants of Food Safety Level in Fruit and Vegetable Wholesalers’ Supply Chain: Evidence from Spain and France

**DOI:** 10.3390/ijerph15102246

**Published:** 2018-10-14

**Authors:** Jesús Hernández-Rubio, Juan C. Pérez-Mesa, Laura Piedra-Muñoz, Emilio Galdeano-Gómez

**Affiliations:** Department of Economics and Business, University of Almería (Agrifood Campus of International Excellence, ceiA3; Mediterranean Research Center on Economics and Sustainable Development, CIMEDES), Ctra. Sacramento s/n, 04120 Almería, Spain; jhr941@ual.es (J.H.-R.); juancarl@ual.es (J.C.P.-M.); galdeano@ual.es (E.G.-G.)

**Keywords:** food safety, fruits and vegetables, wholesaler, importer, long supply chain

## Abstract

Food safety management in short supply chains of fruit and vegetables, controlled by large retailers, has been widely studied in the literature. However, when it comes to traditional long supply chains, which include a greater number of intermediaries and wholesalers who, in some cases, play a dual role as resellers and producers, the mechanisms which promote the use of safety certifications have yet to be clearly defined. The present study intended to fill this gap in the literature and shed light on the food safety level that exists in this channel. In addition, this work attempted to identify the existence of differences between both sales systems. For this purpose, the empirical research studied the most important variables that influence the food safety level of some of the main European fruit and vegetable wholesalers. A survey was thus designed and later applied to Spanish and French intermediaries working in key wholesale markets and in the southeast of Spain, which is the leading commercialization area of these products in Europe. The results revealed the positive influence of specific customers (big retailers) on establishing stricter safety controls within wholesale companies. It was also observed that specific wholesalers also play an important role in the system, namely those dedicated to importing, but which are also responsible for quality and safety inspection of agri-food products exported from third countries into the European Union.

## 1. Introduction

Food safety is considered a public good, as any form of inadequate management could cause severe harm to all participants in the supply chain (i.e., companies and consumers), significantly affecting public health. In addition, food safety obliges stakeholders to collaborate so as to favor the level of transparency needed to guarantee the safety of the products customers consume [1,2]. As regards perishables, the management of supply chains depends on the type and level of collaboration achieved [3,4]. Therefore, differences exist in terms of: (i) Who is ultimately responsible for food safety; (ii) how problems that arise in the supply process are addressed; and (iii) where inspection processes are conducted. Within this context, numerous and varied practices can be observed [5], whether they are conducted by producers [6,7]), public administration [8] or, most notably, big retailers, which are carried out either individually or collectively [9].

**Figure 1:**
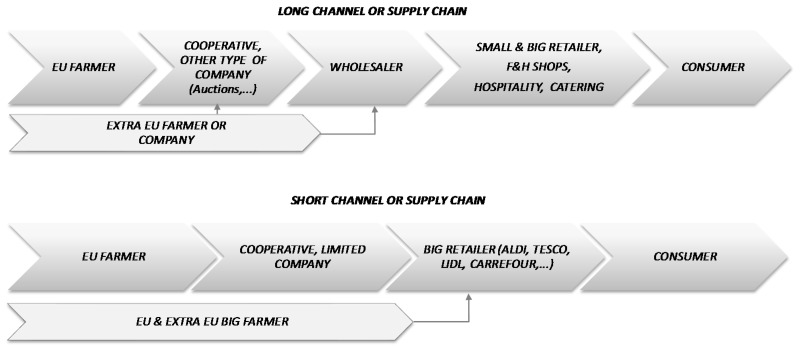
Typology of agri-food supply chains. Source: Own elaboration.

Agri-food short supply chains are controlled by big retailers. By contrast, traditional long supply chains include a greater number of intermediaries and wholesalers, who in some cases play a dual role as resellers and producers (Figure 1).

In the short supply chain, big retailers, or rather their purchasing centers, are the most influential members. In the case of Europe, these companies (e.g., Lidl–Schwarz Gruppe, Aldi, Carrefour, Tesco, Edeka, Rewe) maintain very strict controls. The pressure exerted by these stakeholders to ensure food safety is an aspect which has been studied and verified in the literature [10]. In response to their demands, various private protocols were developed (e.g., GlobalGap, British Retail Consortium-BRC, International Food Standard-IFS, Nurture, Quality Scheme for safe food-QS) to allow retailers to meet the intense requirements of consumers, who had imposed conditions on the rest of the members of the chain. These new processes proved to be even stricter than those of the European regulations in force [11]. Many studies have analyzed how these standards are implemented in the short chain [12,13,14,15].

By contrast, very little attention has been given to this issue in the traditional long supply chain. There are a variety of reasons to explain this lack of interest. The most notable is that the relationships of power are not easily defined due to the existence of multiple stakeholders (producers, wholesalers, and big and small retailers) with rather different market gaps [4]. Consequently, information on how food safety is being managed in this type of supply chain is rather scarce. Given the importance of this matter in the distribution of fresh produce [16], this study seeks to fill this gap to provide possible measures to private operators and public administration so they may safeguard and improve consumer health.

By doing so, the present research aims to determine which factors influence the food safety level (measured by analyzing the percentage of standardized certifications or private protocols) within the long supply chain of fruit and vegetables (F&V) of the main European wholesalers.

Fresh F&V are analyzed because they are one of the most important items in European Union (EU) food trade. In 2017, intra-EU F&V trade accounted for more than 48,300 million euros and extra-EU F&V import represented 25,200 million euros. Moreover, in recent years, F&V have suffered significant food crises that have called into question the controls carried out within the supply chain [17]. In addition, the supply chain of fresh and perishable F&V is heterogeneous and depends on its members, making it is necessary to delve deeper into these scantly studied aspects.

More specifically, the objective of this article is to analyze how the wholesalers’ level (%) of certification is influenced by the degree of customer demand, the type of supplier, and the type of customer. In parallel, the study also aims to gain insight into how these relationships are affected when the main customer is a big retailer.

Additionally, given the differences between EU countries and extra-EU regarding the food safety standards used in production and commercialization processes [18,19,20], this work also seeks to clarify some of the key aspects related to wholesalers of agri-food products exported from third-countries into the EU. If the existence of divergence were detected between the short chain (dominated by the presence of big retailers) and the long chain (where their presence is minor), then we would have to identify import intermediaries as the largest and primary managers of the quality and safety of agri-food products from third-countries to the EU.

For this purpose, a survey is applied to F&V wholesalers in the main Spanish and French markets, as well as those in the leading commercialization area for this type of produce (southeast Spain). These two countries were chosen because they have the largest central wholesale markets in Europe (Mercabarna and Mercamadrid in Spain; and Paris and Saint-Charles in France). Germany is located in the third position (Munich and Hamburg). Spain and France are also important fresh F&V producers and, in addition, along with the Netherlands and the United Kingdom, are two of the main extra-EU F&V importers in Europe (42.63% of total in 2016) [21].

The rest of the article is structured as follows: The next section presents an overview of agri-food safety and how it is implemented according to the type of supply chain; the third section establishes the hypotheses that will be tested; the Methodology section explains the sample, the variables, and the model utilized; the following section presents the empirical results obtained; and, finally, the work closes with the discussion and the most important conclusions.

## 2. Background: Agri-Food Safety in Long Supply Chains

The literature has extensively analyzed food safety related to perishable products, as well as the development and expansion of quality standards, which were primarily the result of social alarm sparked by food alerts during the past decade [22,23].

Further public discussion about the management of food safety could have a positive influence on consumer trust [24,25]. However, given the complexity of the production and supply system, consumers must have faith in chain stakeholders and health safety authorities to compensate for a lack of information and knowledge [26]. The most effective way to increase this trust is to provide consumers with access to information about products, production details, and distribution processes [27,28].

Producers, packing companies, distributors, and retailers all use labeling to emphasize the safety of their products. This practice is carried out voluntarily and/or to comply with laws. In addition, these safety systems have become one of the cornerstones of the supply chain as they synchronize the actions of stakeholders, mainly because competitiveness in the food supply chain is based on the capacity to create intrachannel relationships with a high level of commitment and reliability [29,30].

These systems and standards of food safety can be promoted by both the public and private sectors. In Europe, public regulations and/or obligatory public standards for F&V describe the characteristics that a product must have when it arrives at a certain point in the chain, for example, with no trace of specific substances or with a limited amount of residues [31]. At the same time, private standards have also been established, which are more restrictive and cover aspects that are not regulated by law, above all in terms of materials and processes and even beyond food safety proper (e.g., environmental impact and social responsibility). In Europe, the most widespread private protocols are GlobalGap, BRC, IFS, and QS.

There are numerous factors for establishing these private food safety certifications other than merely complying with legislation [15,32,33]. Some that influence the implementation of these certifications include the need to provide consumers with high-quality products boasting high food safety [15,34,35,36]; to maintain consumer trust [37]; to avoid food crises [17]; to use them as marketing and promotional tools to stand out in the market and improve image and reputation [15,33,36,37]; and to facilitate exportation and access to new markets [32,34,38]. However, the vast majority of studies conclude that the most important and common factor for implementing certifications is that it responds to the requirements of key customers to guarantee safety [15,39,40,41,42]. Therefore, the present work selected the latter as one of the key factors to be analyzed to determine the level of safety certification implementation, along with other aspects related to the supply chain.

Prior to the advent of private safety standards, traditional wholesale transactions were governed only by legal requisites [23]. In this context, there were no differences in levels of food safety between short and long supply chains.

However, given the evolution and predominance of private standards, current food safety measures do differ according to the channel utilized, the characteristics of the channel, the level of collaboration among its members, and customer requirements [3,43,44]. Kleinwechter and Grethe [45] consider that vertical integration is the most important factor when adopting food safety standards. Thus, big retailers have always sought to identify (and even control) where, how, and by whom the fresh products they purchase are produced [46], which, in turn, has given way to direct supply from key growers (Figure 1). Moreover, big retailers have also transferred the management and cost of safety implementation and certification to their suppliers, independently of product origin [47,48,49].

In Europe, in the context of importation and supply chains “governed” by big retailers (i.e., Lidl–Schwarz Gruppe, Aldi, Carrefour, Tesco, Edeka, Rewe), the latter demand private food safety certifications from suppliers, both from third countries and those in the EU. Based on this practice, Okello et al. [50] analyzed African exports of green beans to Europe and they found that it is within this type of chain that European food safety standards are controlled most strictly. In addition, Hou et al. [31] came to a similar conclusion regarding Moroccan F&V exports. Essentially, members of import–export chains from third countries that include big retailers work harder to implement private safety standards. In fact, production in these regions is increasingly similar to that of Europe, primarily because foreign capital is being invested, which in turn favors the transfer of technology and new farming techniques.

By contrast, these certifications are not as well established in traditional long supply chains where big retailers have a smaller presence, even in spite of the large number, scale, and relative importance of wholesalers in relation to F&V consumption. For instance, Table 1 shows that between 34% and 57% of final consumption is supplied through an intermediary.

Despite the growing importance of food safety in long supply chains and the key role played by wholesalers regarding the implementation, management, and commitment to its doctrines, the literature on these subjects is rather scarce (see, for example, [54,55]). In the specific case of F&V, Latouche and Rouvière [56] describe the role and importance of intermediaries within this market, yet they highlight that retailers are more prone to developing private regulations on their own; Rouvière and Latouche [49] indicate that the use of intermediaries and/or importers by supermarket chains constitutes a way of transferring the responsibility to the former if there were to be an error in food safety; Belleáamme and Peitz [57] distinguish between dealers and platforms depending on who owns the product; Rouvière et al. [58] and Rouvière [59] establish a reverse relationship between the size of the intermediary company and the effort made to achieve food safety objectives; García and Poole [60], in an in-depth study, show that wholesalers have developed their own private safety standards (unstandardized) based on different regulations, customer demands, and their own guidelines.

While these works address the F&V sector, none of them directly addresses wholesalers and their role as food safety managers. Therefore, a gap exists in the literature for an analysis of the importance and responsibility of the aforementioned group concerning this matter, which the current study intends to explore. Furthermore, it is possible that the trend towards short-chain supply, which minimizes the role of intermediaries, is leading wholesalers to seek out ways of adding value to products. Essentially, the latter may be going beyond the safety requirements of current standards by creating their own brands and labeling, effectively making food safety their own priority.

## 3. Hypotheses of Analysis

As highlighted in the previous section, operators in the chain are motivated to fulfill private food safety certifications to obtain numerous benefits [61], which include improving product quality and safety [35,36], improving company image and reputation [33,37], or gaining access to market [62,63]; but, above all, legal mandate [64] and pressure from customers [15,39,40,41,42]. With regard to customers specifically, different drivers of certification level are addressed in the literature, such as the following: Meeting customer requirements or demands [15,32]; anticipating future customer requirements [40]; satisfying customers [65]; achieving customer awareness of food safety [42]; retaining existing customers and/or attracting new customers [39,40]; reducing customer complaints [39,40]; passing customer audits/inspections [39,40]; and lowering the risk of compromising food safety for customers [42].

In short, after compulsory fulfillment of legal requirements, most of the literature positions customer requirements as the second factor for companies to implement private certifications. In this context, the present study proposes the following hypothesis with regard to F&V wholesalers:

**Hypothesis** **H1.** *The wholesaler’s certification level depends on the intensity of their customers’ requirements.*

The advent and tremendous growth of supermarket chains in recent decades has shaped the evolution of standards that did not previously exist. Furthermore, said standards are only completely fulfilled in supply chains where they are in force [60,66]. Big retailers often seek to identify and control where, how, and by whom the fresh products they purchase are produced [46]. Thus, in supply chains where supermarket chains have an influence, food safety standards are controlled more strictly [31,50]. In these cases, it is more likely that supermarkets detect unsafe products and that wholesalers increase their safety measures to safeguard themselves from this risk [9]. The mere presence of big retailers in the supply chain increases pressure on all other members to guarantee food safety [10], imposing far stricter conditions than those of existing European Community regulations [11]. More specifically, Rouvière et al. [58] established that there is a direct positive relationship between the extent of the effort made by wholesalers regarding food safety and the fact that big retailers are the primary customers of the former. If, on one hand, customer requirements are the key determining factor behind certification, and, on the other, big retailers are the members of the chain that exert the greatest pressure to guarantee food safety, it follows that the demand for wholesalers to certify their products must be even greater if their main customers are big retailers. In this line, the following hypotheses are presented:

**Hypothesis** **H2.** *The wholesaler’s certification level is greater when they sell to big retailers.*

**Hypothesis** **H3.** *The level of sales to big retailers increases the relationship between a wholesaler’s certification level and the intensity of their customers’ requirements.*

Private food safety certifications are considered to be obstacles to commerce and, on occasions, a disadvantage for developing countries [9]. As can be seen in Figure 1, European F&V wholesalers are supplied by European farmers and cooperatives (which are even producers themselves on some occasions) and imports from third countries outside of Europe. In the literature, it is common to attribute poorer quality and safety to imported produce. Moreover, according to [56], the safer the product, the lower its share of imports made indirectly through wholesalers will be, as opposed to directly through retailers. If this relationship were to be corroborated, it would be logical for wholesalers that deal primarily in imported produce (from outside the EU) to have a lower level of certification. In this context, the following hypotheses are presented:

**Hypothesis** **H4.** *Wholesalers that are primarily importers have a lower level of certification.*

**Hypothesis** **H5.** *The type of wholesale company (dealing mainly in imports) reduces the relationship between level of certification and the intensity of customer requirements.*

**Figure 2:**
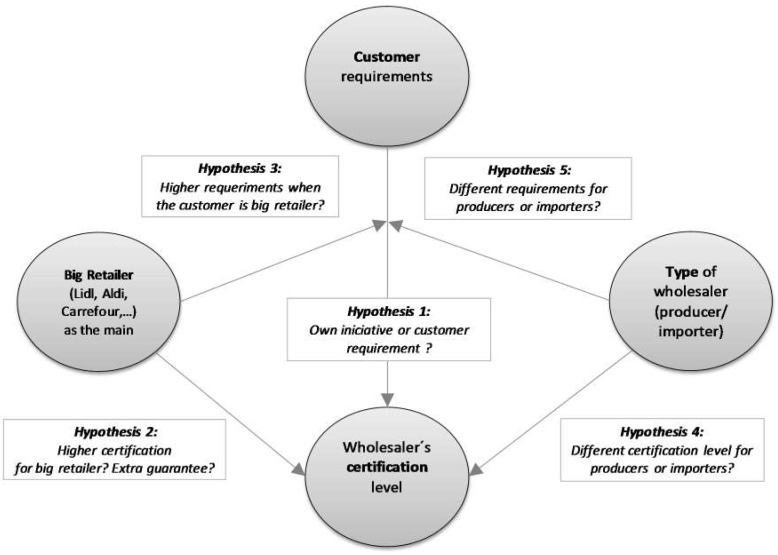
Tested relationships.

Figure 2 summarizes the relationships and hypotheses analyzed.

## 4. Methodology

### 4.1. Data

The sample utilized for the empirical analysis comprised 102 wholesalers located in Spain and France. A convenience survey was performed to obtain a representative sampling. Forty-two surveys were conducted in Spain and 60 in France, accounting for 51.3 and 54.4%, respectively, of extra-EU F&V imports of the country, and approximately 20% of total extra-EU F&V imports in terms of the sales volume, according to Eurostat [21]. In the case of Spain, the two largest wholesale centers (*Mercas*) in the country were included, namely Mercamadrid and Mercabarna, along with key wholesalers located in the three main fruit and vegetable production areas (Almeria, Murcia, and Valencia; all located in the southeast of Spain). With the aim of strengthening the sample, other areas were included, such as Granada, Huelva, and Castellon. As for France, its two largest wholesale centers were also included: Saint Charles (Perpignan) and Rungis (Paris). No significant differences were observed in the operations of wholesalers with regard to country of origin. Both countries featured large and small businesses and enough diversity to determine the effect of the different variables used in the model on food safety level.

These wholesalers were surveyed to assess the status of their food safety practices by means of a structured questionnaire with three sections. The first covered basic company information, such as name, number of employees, turnover, role in the supply chain, top imported product, and importation method. Regarding food safety management, the second section dealt with the company’s suppliers and upstream relationships, while the third was used to extract information about their customers and downstream relationships. In most of the questions, the participants were requested to select the appropriate answers from a list of options. The surveys were conducted by means of personal interviews with the 102 companies. This sample represents a total turnover of 2563 million euros and 2,871,649 tons of imports in 2015.

These wholesalers are typically small and medium-sized intermediary companies (in some cases they possess their own production), which comprise the traditional long supply chain. This chain existed as the dominant sales systems prior to the advent of big retailers, a transition which occurred in the latter half of the 1990s. Intermediary companies have not received the same pressure to implement safety standards to the same extent as local producers and marketing companies whose customers are big retailers in the short chain.

**Figure 3:**
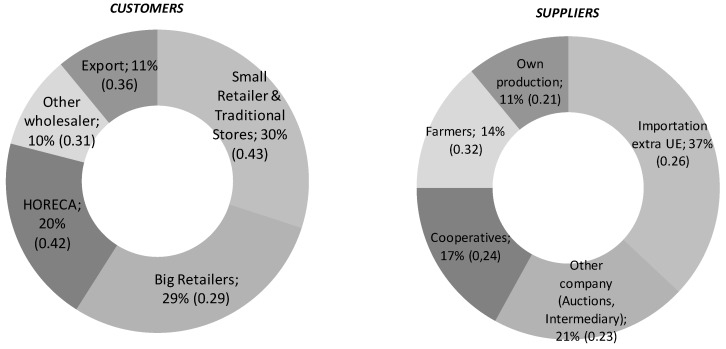
Customers and suppliers of wholesalers. Notes: HORECA stands for Hotels, Restaurants and Catering. In parentheses, standard deviation.

Figure 3 displays the customers and suppliers of the wholesale companies interviewed. The characteristics of the customers vary, but traditional stores and small retailers constitute a considerable percentage, which is why these intermediaries continue to have strong links with their traditional customers. Nevertheless, big retailers stand out as significant customers. Among the suppliers, the most important is importation, followed by origin-based intermediary companies. Taking into consideration the percentages of certification in the sample analyzed, 42% of F&V is certified on average. Specifically in terms of customers, big retailers’ percentage of certified products is 79%, that of traditional stores, small retailers, and HORECA stands at 42%, and that of all other wholesalers and importers totals 24%.

### 4.2. Variables and Model Description

For the empirical analysis, a hierarchical regression model was used as follows:

CERT = f (CUST, DIST, TYPE, X_Control_) (1)

where the dependent variable (CERT) is the percentage of total production sold by the wholesaler that has some kind of safety certification (ISO, GlobalGap, BRS, IFS, or other).

The independent variables are the following:

- CUST: The degree of customer requirements measured by a 1–5 Likert scale where 1 is low customer demand and 5 is maximum requirement. More specifically, this variable represents the perception of wholesalers regarding the pressure exerted on them by their main customers in terms of food quality and safety [15,58]. The intensity of this variable depends on a vast range of factors, such as the importance that customers place on certification, in-house safety control (without third parties), the level of complaints, and even the degree of trust between wholesalers and customers [42].
- DIST: Represents the type of customer or distributor. It is the percentage of sales carried out through big retailers over total sales. According to [58], there is a direct relationship between a wholesaler’s level of certification and the fact that their main customer is a big retailer.
- TYPE: Indicates the type of supplier, that is, whether the wholesaler is primarily an importer (of F&V from third countries to the EU) or is mainly supplied by their own production, cooperatives, or European farmers [56]. This is a dummy variable that takes a value of 1 when the majority of the wholesaler’s produce comes from importation, and 0 in all other cases.

The last two variables (DIST and TYPE) are also incorporated as moderators to determine how types of customer and supplier affect the relationship between certification and customer requirements. In addition, the following control variables are introduced:

- AGE: The number of years that the wholesaler has been operating as an indicator of experience and years in the business.
- TURN: The annual turnover of the wholesaler (in thousands of euros). It is an indicator of company size. In the literature, the size of a company produces varied results in relation to efforts made to implement food safety. Some studies [67,68] indicate that the largest companies are those which make the greatest efforts in this regard, while others [58] suggest the opposite.
- EMP: The number of employees, which is also indicative of the size of the wholesaler.
- DIV: The degree of specialization, measured by the number of products the wholesaler deals in. The diversification of a product portfolio might prevent a company from receiving certification.

Table 2 displays the description of the variables used in the analysis. It is particularly noteworthy that the average level of certification in the sample does not reach 50%. In addition, the degree of customer requirements is low and fails to reach the average value. Only 26% of the sample can be considered as primarily importers. As for big retailers, they represent a small percentage of total sales (29%). In general, the sample contains companies with a substantial turnover (over 40 million euros), but there is also very high heterogeneity among them. In terms of the amount of time companies have been operating in the sector, the figure reaches nearly 30 years. In terms of diversification, companies commercialize eight different products on average; however, some companies are completely specialized. 

By taking logarithms, except for the dummies, the final equation for estimating is the following:

lnCERT_i_ = β_0_ + β_2_CUST_i_ + β_3_lnDIST_i_ + β_4_TYPE_i_ + β_5_(lnDIST_i_ × lnCUST_i_) + β_6_(TYPE_i_ × lnCUST_i_) + β_7_lnAGE_i_ + β_8_lnTURN_i_ + β_9_lnEMP_i_ + β_10_lnDIV_i_ + ε_i_ (2)

where ε_i_ is an error term.

## 5. Results

Table 3 presents the results of the hierarchical regression of Model (2) and the diagnostic tests. The three-stage forward stepwise regression was used in the analysis for the robustness check [69]. According to the results of the estimations, there is a significant relationship between the degree of customer requirements (CUST) and the level of certification (CERT), which indicates that the wholesaler implements the requirements of their customer, that is, they obey the customer’s instructions. This confirms Hypothesis 1. This relationship also demonstrates that the wholesaler does not act on their own initiative with regard to certification; instead, they act in accordance with the attitude of the customer.

As for Hypothesis 2, it is confirmed that the variable DIST is significant and that it positively influences the level of certification. Therefore, Hypothesis 2 is also confirmed, meaning the percentage of sales made to big retailers affects the level of certification of wholesalers. This situation is logical given that this type of customer requires an extra guarantee of safety as they assume the risk of selling a generic product under their brand.

**Figure 4:**
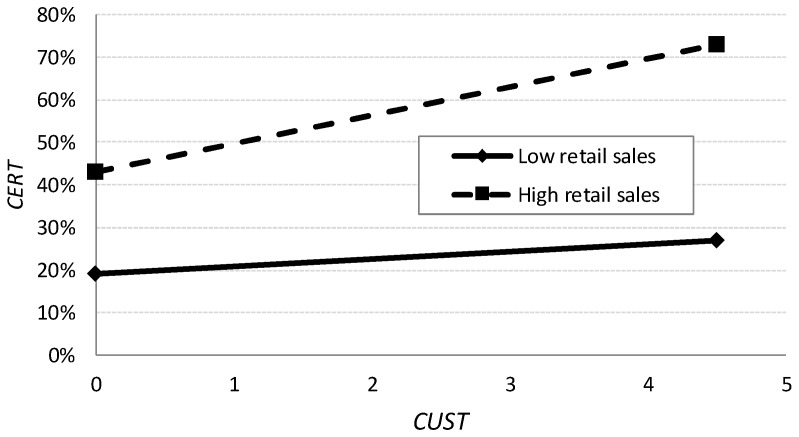
Interaction effects *DIST* × *CUST.* Notes: *CERT* is the percentage of certified production sold by the wholesaler; *CUST* is the degree of customer requirements, where 1 is lowest and 5 is maximum; *DIST* is the percentage of sales carried out through big retailers.

To interpret the multiplied effects, we use the graphic procedure proposed by the authors of [70]. In Figure 4, “high” values indicate a standard deviation above the mean, whereas “low” values indicate a standard deviation below the mean. Thus, the significance of the multiplicative variable DIST × CUST is relevant. Precisely as shown in Figure 4, this implies that sales through big retailers (DIST) increase the relationship (slope) between the degree of customer requirements (CUST) and certification (CERT). In other words, the degree of obedience to the customer is greater when the latter is, for the most part, a big retailer. Hypothesis 3 is therefore corroborated.

With regard to wholesalers which are primarily importers (TYPE), although this variable displays the expected sign, said status bears no statistically significant influence on the certification of the wholesaler. In other words, the produce commercialized has the same level of safety, regardless of whether it came from third countries, whether it is the company’s own production or is purchased within the EU. As a result, Hypothesis 4 is not confirmed.

**Figure 5:**
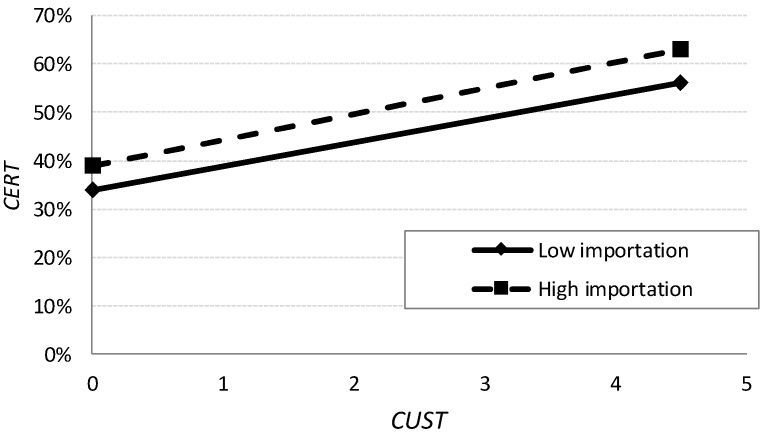
Interaction effects *TYPE* × *CUST.* Note: *CERT* is the percentage of certified production sold by the wholesaler; *CUST* is the degree of customer requirements, where 1 is lowest and 5 is maximum; *TYPE* indicates where the majority of the wholesaler’s produce comes from importation.

The interaction TYPE × CUST is not significant either, indicating that dealing chiefly in importation does not make the customer exert more pressure on the wholesaler to increase their certification due to lower trust in the origin of the product. Figure 5 also shows that operating mainly as an importer (TYPE) does not substantially modify the relationship (slope) between degree of customer requirements (CUST) and certification (CERT). This reveals that, for the customer, imported produce currently receives the same treatment as European production. Thus, Hypothesis 5 is not confirmed.

With regard to the control variables, it is noteworthy that a company’s time in operation (AGE) has a negative influence on the level of certification. This aspect seems to indicate that companies with traditional business structures and clearly defined channels (and presumably stable relationships with customers) do not need to certify their produce to guarantee its safety. As for level of turnover, number of employees, and business diversification, none of these factors display any relationship with the percentage of certified produce.

## 6. Discussions

The present work analyzes the main factors that influence the food safety levels of wholesalers in the traditional long supply chain of F&V. This study was based on a sample comprising 102 intermediaries operating in the key wholesale market centers in Spain and France, as well as in the most important Spanish production areas. The food safety level itself was measured as the percentage of total production sold by wholesalers that possesses some safety certification, which, on average, registers at around 42%.

According to the estimations made, firstly, a significant positive relationship was found between the degree of customer requirements and the level of certification. This aspect indicates that wholesalers implement the requirements of their customers regarding matters of quality and food safety. This result is in line with the majority of the literature, which identifies customer pressure as the main factor why companies implement private certifications after having already fulfilled legal regulations [15,32,39,40,41,42]. In this regard, it could be said that intermediaries do not act on their own initiative; instead, they follow legal requirements and those of their customers.

Secondly, the level of certification of intermediaries is also influenced by the type of customer. In the present case, the most common customer (30% of sales) is of the traditional type, namely small retailers and traditional stores. Nevertheless, big retailers are close behind, representing 29% of sales. It was possible to confirm that the inclusion of this type of company in the chain positively influences the level of certification of the intermediary, thereby guaranteeing higher safety. This result is in accordance with those found in other works such as those in References [10] and [58]. In turn, sales through big retailers increase the relationship between the degree of customer requirements and certification. In other terms, the wholesaler responds to pressures from their customer to a greater extent (“obeys”) when the latter is a big retailer. It is thus confirmed that the latter acts as a hub within the chain and effectively dominates the relationships in it. Said level of requirement relaxes when percentages of sales to big retailers decrease.

Thirdly, it was also determined that the food safety level of the wholesaler does not significantly depend on the type of supplier. According to the data analyzed, the most common suppliers of these wholesalers are companies or farmers from third countries that do not belong to the EU (37%), followed by intermediary companies located in production areas. However, level of certification is not significantly affected by the fact that the wholesaler is a producer or not also an intermediary, and by the type of supplier (primarily importation). The customer will impose the same level of requirement for both European and imported produce. Customers see the intermediary as interlocutors and charge them with all responsibility regarding matters of safety, regardless of the origin of the produce. This aspect is contradictory to the belief that imported produce is of lesser quality and safety, above all that which has passed through wholesalers [56].

Finally, the level of certification of an intermediary is negatively influenced by the number of years it has been in operation. In general, we are dealing with companies that have been in the sector for a long period of time (29 years on average), which could explain why their produce does not need to be certified in order to guarantee safety to their customers, simply because relationships of trust prevail in these situations. In addition, the size of companies and their business diversification do not influence the food safety level.

## 7. Conclusions

It can be concluded that the certification of wholesalers is moderate. Therefore, the long supply chain of F&V in Europe, in which a big retailer is not the main customer, displays low levels of certification compared to those of the short chain. In those cases where big retailers have a dominant presence as the main customer in the long chain, their vast power to demand certified produce is clearly observed. In addition, wholesalers dependent on importation do play an important role as managers of the quality and safety of agri-food products from third countries to the EU. Given that within the borders of the EU there are minimum standards, the role of wholesalers is pivotal when they operate as importers from third countries with regulatory frameworks and markets that differ from those of Europe. Thus, the role of the importer-wholesaler becomes an important hub for the food safety of produce that will be consumed in the EU.

These results have several implications for policies. On the one hand, it is necessary to continue to promote programs that foster the voluntary implementation of safety certifications by wholesalers as a differentiation strategy with respect to the competition. On the other hand, it is clear that the legislation governing this chain is a priority with regard to safety control of imports into Europe. 

The subject of the intermediary channel in fresh F&V distribution is of great importance. Although it is scarcely analyzed in the literature, the present work contributes to this topic by providing evidence on food safety from the point of view of wholesalers. Nevertheless, this study is not without its limitations, which could be used as reference for future research. For example, given that the present work focuses on data for only one year, subsequent studies could attempt to identify the evolution of these factors over a longer period of time. Moreover, it would be useful to conduct a comparison with wholesalers located in other countries and regions. In addition, future works could expand the factors that have an influence on the food safety level of intermediaries, thereby delving deeper into the differences between the various types of supply chains and incorporating consumer perception on this matter and how the trend towards more direct supply chains can influence the operations of intermediaries.

## Figures and Tables

**Table 1:** Fruit and vegetable (F&V) purchase percentage of end consumer and percentage of supply from wholesalers or intermediaries (2014).

Consumer Direct Purchase ^1^	Spain	France	Germany
Super-Hyper-Discount	43% (20%)	73% (35%)	86% (25%)
Traditional Shops	40% (95%)	20% (90%)	12% (92%)
Others (Self-consumption, street markets)	17% (63%)	7% (83%)	2% (90%)
Total	100% (57%)	100% (49%)	100% (34%)

^1^ In parenthesis, percentage of supply from wholesalers. Row ‘Total’ shows the weighted mean percentage of final consumption that is supplied through a wholesaler or intermediary. Source: Own elaboration based on data from [51,52,53].

**Table 2:** Description of variables and correlation.

	*PROM*	*DESV*	*MAX*	*MIN*	*CERT*	*CUST*	*DIST*	*TYPE*	*TURN*	*AGE*	*EMP*
*CERT*	42.30	34.34	100.00	0.00	1						
*CUST*	2.43	1.45	5.00	1.00	0.160	1					
*DIST*	28.89	27.42	90.00	0.00	−0.102	0.181	1				
*TYPE*	0.26	0.44	1.00	0.00	0.210	0.426	0.215	1			
*TURN*	40,738	83,498	755,851	180.00	0.017	0.307	0.119	0.170	1		
*AGE*	29.05	17.43	51.00	3.00	−0.194	0.074	0.061	0.043	0.372	1	
*EMP*	25.38	42.19	301.00	1.00	−0.061	0.130	0.049	−0.029	0.686	0.395	1
*DIV*	8.46	6.85	17.00	1.00	0.102	0.098	0.191	0.038	0.321	0.033	0.271

**Table 3:** Estimated model (Dependent variable *CERT* = percentage of certified product).

Variable	Model 1	Model 2	Model 3
Constant	−0.373	−0.424	−0.321
*CUST* (Hyp. 1)	0.465 **	0.457 **	0.387 **
*DIST* (Hyp. 2)		0.268 *	0.305 *
*TYPE* (Hyp. 4)		−0.145	−0.237
*DIST* × *CUST* (Hyp. 3)			0.109 *
*TYPE* × *CUST* (Hyp. 5)			0.018
AGE	−0.022 **	−0.030 **	−0.012 *
TURN	0.060	0.063	0.018
EMP	0.024	0.015	0.005
DIV	0.002	−0.008	−0.011
*R^2^*	0.338	0.453	0.618
Variation R^2^	0.115	0.165	0.115
*Adjusted R^2^*	0.295	0.434	0.455
*D-W*	1.919	1.615	1.558
χ^2^ Farrar-Glauber	11,721	16,009	19,565
White Test	6382	9482	7410

* and ** denote significance at 10 and 5 percent level, respectively. All the variables are transformed into logarithms except for the dummies.
